# Endogenous melatonin and oxidatively damaged guanine in DNA

**DOI:** 10.1186/1472-6823-9-22

**Published:** 2009-10-18

**Authors:** Zoreh Davanipour, Henrik E Poulsen, Allan Weimann, Eugene Sobel

**Affiliations:** 1 Preventive Medicine, Feinberg School of Medicine, Northwestern University, Chicago IL 60611, USA; 2 Pharmacology, Rigshospitalet, Copenhagen, Denmark; 3 Friends Research Institute, Los Angeles CA 90064, USA

## Abstract

**Background:**

A significant body of literature indicates that melatonin, a hormone primarily produced nocturnally by the pineal gland, is an important scavenger of hydroxyl radicals and other reactive oxygen species. Melatonin may also lower the rate of DNA base damage resulting from hydroxyl radical attack and increase the rate of repair of that damage. This paper reports the results of a study relating the level of overnight melatonin production to the overnight excretion of the two primary urinary metabolites of the repair of oxidatively damaged guanine in DNA.

**Methods:**

Mother-father-daughter(s) families (n = 55) were recruited and provided complete overnight urine samples. Total overnight creatinine-adjusted 6-sulphatoxymelatonin (aMT6s/Cr) has been shown to be highly correlated with total overnight melatonin production. Urinary 8-oxo-7,8-dihydro-guanine (8-oxoGua) results from the repair of DNA or RNA guanine via the nucleobase excision repair pathway, while urinary 8-oxo-7,8-dihydro-2'-deoxyguanosine (8-oxodG) may possibly result from the repair of DNA guanine via the nucleotide excision repair pathway. Total overnight urinary levels of 8-oxodG and 8-oxoGua are therefore a measure of total overnight guanine DNA damage. 8-oxodG and 8-oxoGua were measured using a high-performance liquid chromatography-electrospray ionization tandem mass spectrometry assay. The mother, father, and oldest sampled daughter were used for these analyses. Comparisons between the mothers, fathers, and daughters were calculated for aMT6s/Cr, 8-oxodG, and 8-oxoGua. Regression analyses of 8-oxodG and 8-oxoGua on aMT6s/Cr were conducted for mothers, fathers, and daughters separately, adjusting for age and BMI (or weight).

**Results:**

Among the mothers, age range 42-80, lower melatonin production (as measured by aMT6s/CR) was associated with significantly higher levels of 8-oxodG (p < 0.05), but not with 8-oxoGua. Among the fathers, age range 46-80, lower melatonin production was associated with marginally higher levels of 8-oxoGua (p < 0.07), but not with 8-oxodG. Among the daughters, no relationship was found between melatonin levels and either 8-oxodG or 8-oxoGua levels. When the mother and father data were further analyzed using only subjects older than the oldest daughter, the associations became somewhat stronger.

**Conclusion:**

Low levels of endogenous melatonin production among older individuals may lead to higher levels of oxidatively damaged guanine in DNA, thereby possibly increasing the risk of developing cancer. The possible different effects of melatonin in the rates of utilization of pathways for repair of oxidatively damaged guanine in DNA identified between older women and older men are intriguing.

## Background

Melatonin is a powerful scavenger of reactive oxygen species (ROS), particularly the hydroxyl radical (^•^OH), at both physiologic and pharmacologic concentrations [1]. In the literature, "physiologic" refers to normal blood level concentrations of melatonin, while "pharmacologic" indicates 2-3 orders of magnitude higher concentration. Recently, intracellular levels of melatonin, especially within the nucleus, have been shown to be naturally at "pharmacologic" levels for all cellular organelles studied to date [2,3]. Melatonin may thus play an active role in the reduction of the risk of breast and other cancers associated with oxidatively damaged DNA.

Guanine bases in DNA and RNA are particularly susceptible to damage from ^•^OH [4,5]. Urinary 8-oxo-7,8-dihydro-2'-deoxyguanosine (8-oxodG) and 8-oxo-7,8-dihydro-guanine (8-oxoGua) result from the repair of oxidatively damaged guanine in DNA [6]. Current evidence suggests that neither diet nor cell death/turnover is a significant contributor to urinary 8-oxodG or 8-oxoGua [7]. 8-oxoGua, the oxidized guanine base in either DNA or RNA, results from base excision repair (BER). 8-oxodG, the oxidized guanine base in DNA with the sugar, may possibly result from nucleotide excision repair (NER) [7-11]. Oxidation and subsequent repair within the nucleotide pool is also possible [7]. However, the extent of the contribution of these two repair processes is unknown.

This study investigates the relationships between the total overnight accumulation of urinary aMT6s/Cr and the total overnight accumulation of urinary 8-oxodG and 8-oxoGua. aMT6s is the primary urinary metabolite of melatonin. Total overnight urinary aMT6s/Cr is highly correlated with the total overnight melatonin production [12,13]. To determine total overnight melatonin production, blood samples are taken, say, every hour overnight and the blood level of melatonin is determined. This allows for the estimation of the total amount of melatonin produced overnight by an area under the curve technique because the melatonin is rather quickly either metabolized or stored within cells [12,13]. Thus, assay of total overnight urinary aMT6s/Cr is a much more feasible method of estimating total overnight melatonin production.

It was therefore hypothesized that lower levels of melatonin production would be associated with higher levels of 8-oxodG and 8-oxoGua.

## Methods

Urine samples, stored at -20°C for less than 1-2 years, were available from an earlier cross-sectional study designed to model the relationship between mother and daughter levels of melatonin production, after adjusting the daughter's melatonin level for the father's melatonin level.

### Subjects

Fifty-five (55) nuclear biologic families were recruited in an earlier cross-sectional study designed to model the relationship between mother and daughter levels of melatonin production, after adjusting the daughter's melatonin level for the father's melatonin level. The mothers, fathers, and daughters had no personal history of cancer and were apparently in reasonable health for their age. No subject was taking a melatonin supplement. The families were from western New York, USA. They were recruited primarily through notices and fliers.

### Questionnaire Data

Epidemiologic data were obtained via a structured questionnaire. Data items included date of birth, ethnicity, weight, height, complete occupational history, use of electrical or electronic equipment, hobbies, exercise, reproductive history (women only), medical history, diet, and use of prescription and over-the-counter supplements. For this study, weight, body mass index (BMI), exercise, smoking, alcohol use, red meat consumption, parity, and spontaneous abortion history were considered in the statistical analyses.

### Urine Samples

Each participating member of a nuclear family provided a complete overnight sample. Subjects were instructed not to collect any urine prior to going to bed and to collect all voided urine after going to bed and the first void in the morning upon getting up. No effort was made to have the subjects go to bed at a particular time or get up at a particular time. For each member of a family unit, the urine collections were within 3 days of one another. Thus, seasonal variation of melatonin production within families is not an issue. Urine collection equipment was provided along with detailed instructions, which were reviewed with each subject. Urine samples were stored in the subject's refrigerator prior to the morning pick up or delivery of the urine to a study staff member. The volume of each sample was measured, aliquots were made and stored at -20°C, and a written record of each sample was kept.

### Laboratory Assays and Data

Urine samples were stored at -20°C for less than 1-2 years. aMT6s was assayed using the kits from IBL Immuno-Biological Laboratories, Hamburg, Germany. Creatinine was measured using the *Vitros *CREA Slide (Ortho-Clinical Diagnostics, Inc.) method. These assays were performed by a laboratory at Roswell Park Cancer Institute. 8-oxodG and 8-oxoGua were measured simultaneously using high performance liquid chromatography-electrospray ionization tandem mass spectrometry (HPLC-ESI) [5]. The units of measurement are as follows: aMT6s - ng/ml; creatinine - mg/ml; 8-oxodG and 8-oxoGua - nmol/litre. Upon multiplication by the overnight volume of urine, the units are ng, mg and nmol. The 8-oxodG and 8-oxoGua assays were performed in Dr. Poulsen's laboratory in Denmark.

Total overnight 8-oxodG and 8-oxoGua were not adjusted for total creatinine, as is commonly done for spot samples but not 24-hour urine samples. Poulsen et al. (1998) found that the Pearson correlation between total 24-hour urinary 8-oxodG accumulation and an independent creatinine-adjusted morning spot measurement was only 0.5 [14]. In the present study, the Pearson correlations between total overnight accumulation and creatinine-adjusted 8-oxodG from the same urine sample were 0.52 (mothers), 0.66 (fathers), and 0.45 (oldest daughters). These are close to the Poulsen et al. finding. For 8-oxoGua, the correlations were 0.90 (mothers), 0.90 (fathers), and 0.72 (oldest daughters) in the present study. While the data to determine the correlations between complete overnight and 24-hour 8-oxodG and 8-oxoGua accumulation are not available, it seems reasonable that complete overnight urine samples (unadjusted) are more highly correlated with 24-hour urine samples (unadjusted) than are creatinine-adjusted complete overnight urine samples.

Measurement of DNA oxidation in extracted DNA is essentially 100% successful using chromatography. However, measurement in urine is a challenge, Some urine samples contain interfering substances, and it is thus not always possible to avoid overlapping peaks in the chromatography [5]. Immunologically-based assays present even greater problems. Presumably, in the years to come, column and pump technology in chromatography will improve and will make it possible to overcome these problems. Until then, missing results cannot be avoided. Thus, the number of samples for 8-oxodG and 8-oxoGua with valid data is lower than 55 within the mother, father, and oldest daughter groups due to some chromatographic assay problems. Specifically, assay problems resulted in the loss of 9% - 18% of the 8-oxodG data and 13% - 27% of the 8-oxoGua data among the mothers, fathers and oldest daughters. The limit of detection for both 8-oxodG and 8-oxoGua is approximately 0.5 nanomoles (nmol) per litre.

### Statistical Analyses

In the analyses, the data for the mother, father, and oldest sampled daughter were used because this model is more appropriate for use in future epidemiologic studies. Paired t-tests were performed to compare aMT6s/Cr, total 8-oxodG, and total 8-oxoGua between mothers, fathers, and the daughter, in a pairwise manner. Using paired t-tests accounts for possible correlations between the family members. The relationships between (1) overnight urinary aMT6s/Cr and (2) total urinary overnight 8-oxodG and 8-oxoGua were derived using regression analyses, with age and BMI (or weight) as the covariates. A p-value equal to or below 0.05 was required for statistical significance, while "marginal significance" was defined as a p-value less than or equal to 0.10, but greater than 0.05. Analyses were carried out on mothers, fathers and the daughter separately. Two outliers, defined as a value 3 times larger than the next largest value, were eliminated from the father's regression analyses because of their extreme influence on the results. Additional analyses were carried out with only those mothers and fathers who were older than any daughter, in order not to have an overlap in the ages of the two generations. The oldest daughter was 51.6 years old. SAS statistical software was used [15].

### Informed Consent and Institutional Review Board Approval

This study was approved by the Institutional Review Board at Friends Research Institute and the United States Department of Defense Congressionally Directed Medical Research Program. Each participant signed a written consent form. The study complied with the Declaration of Helsinki.

## Results

Inclusion of current exercise, smoking, alcohol consumption, red meat consumption, parity, and spontaneous abortion history in the analyses did not affect the relationships between creatinine-adjusted aMT6s and 8-oxodG or 8-oxoGua (data not shown). They are therefore not included in the results discussed below.

Table 1 provides the descriptive statistics for subjects by status: mother, father, and daughter. The age ranges (years) were similar for the mothers and fathers: 43.5 - 80.7 and 46.1 - 80.9, respectively. The age range for the daughters was 18.6 - 51.6. The mean ages were as follows: mothers - 59.6; fathers - 62.4; and daughters - 32.9. Mean 8-oxoGua urinary excretion levels were 6-8 times that of mean urinary 8-oxodG levels. The fathers' aMT6s/Cr values were generally lower than the mothers' values, although the range was nearly identical. For example, the 25^th^-75^th ^percentile ranges were different: 33.3 - 80.3 for the mothers and 15.6 - 49.9 for the fathers.

**Table 1:** Descriptive Statistics by Mothers, Fathers, and Daughters

**Variable**	**Mothers**	**Fathers**	**Daughters**
**AGE (years)**			
N	55	55	55
Mean	59.6	62.4	32.9
Standard	10.2	10.5	9.8
Deviation			
25^th ^- 75^th^	50.2-68.4	52.9-71.6	24.3-38.4
Percentile			
Min - Max	43.5-80.7	46.1-80.9	18.6-51.6

**BMI**			
N	55	55	55
Mean	25.8	27.3	25.4
Standard	4.6	4.7	6.3
Deviation			
25^th ^- 75^th^	22.7-28.8	24.4-30.7	21.5-27.5
Percentile			
Min - Max	19.5-40.2	18.5-39.6	18.5-52.9

**Weight (kg)**			
N	55	55	55
Mean	69.0	87.4	68.4
Standard	11.7	16.9	19.1
Deviation			
25^th ^- 75^th^	59.4-74.8	76.2-97.5	56.2-72.6
Percentile			
Min - Max	48.5-104.3	56.7-136.1	47.6-162.4

**Creatinine-Adjusted aMT6s (ng/mg)**			
N	55	55	55
Mean	55.5	36.0	79.6
Standard	30.0	26.6	47.9
Deviation			
25^th ^- 75^th^	33.3-80.3	15.6-49.9	45.1-100.1
Percentile			
Min - Max	4.0-123.2	4.3-123.2	6.1-240.8

**8-oxodG (nmol)**			
N	50	45	47
Mean	4.7	6.7	5.9
Standard	2.1	3.5	4.6
Deviation			
25^th ^- 75^th^	2.9-6.2	4.2-7.9	3.6-5.9
Percentile			
Min - Max	1.1-9.3	0.6-15.9	1.5-26.7

**8-oxoGua (nmol)**			
N	44	40	48
Mean	33.9	54.4	39.7
Standard	34.6	65.9	46.8
Deviation			
25^th ^- 75^th^	14.2-36.7	20.4-61.2	15.5-42.5
Percentile			
Min - Max	4.3-173.8	4.6-331.2	4.8-267.3

For puposes of possible comparison with published studies which used creatinine-adjusted 8-oxodG and/or 8-oxoGua values, the creatinine-adjusted means (standard deviations) in nmol/mmol are as follows: (1) 8-oxodG: Mothers - 1.5 (0.53); Fathers - 1.3 (0.64); Oldest Daughters - 1.3 (0.49); (2) 8-oxoGua: Mothers - 11.7 (11.43); Fathers - 10.3 (10.68); Oldest Daughters - 9.0 (8.17).

Table 2 provides pairwise comparisons of aMT6s/Cr, 8-oxodG, and 8-oxoGua values between mothers, fathers, and daughters. The mothers and the daughters had significantly higher mean aMT6s/Cr values than the fathers. In addition, the daughters had a significantly higher mean aMT6s/Cr than their mothers. Fathers had a significantly higher urinary 8-oxodG mean than did the mothers (p < 0.001), while their mean 8-oxoGua level was marginally (p = 0.08) higher.

**Table 2:** Paired Comparisons of Mothers, Fathers, and Daughters: Creatinine-Adjusted aMT6s, 8-oxodG, and 8-oxoGua.

**Variable**	**Comparison**	**N**	**Ratio**	**Mean Difference**	**St. Error**	p-Value*
**Creatinine-**	M vs F	55	1.54	19.5	5.6	0.001
**Adjusted**	D vs M	55	1.43	24.0	6.2	0.0003
**aMT6s**	D vs F	55	2.21	43.5	6.1	<0.0001

**8-oxodG**	F vs M	40	1.42	2.0	0.5	<0.001
	D vs M	45	1.26	1.3	0.8	0.11
	F vs D	37	1.14	0.8	0.6	0.20

**8-oxoGua**	F vs M	31	1.60	20.5	11.3	0.08
	D vs M	40	1.17	8.0	8.7	0.37
	F vs D	33	1.37	8.9	15.8	0.58

* The paired t-test was used. Pairing was the reason for the decrease in sample sizes. There was little difference when unpaired t-tests, with Cochran's adjustment for unequal standard deviations, were used for the analysis.

Pearson correlations were also calculated between urinary 8-oxodG and 8-oxoGua for the mothers, fathers, and daughters, separately. The correlations were, respectively, 0.10 (p = 0.50), 0.19 (p = 0.26), and 0.71 (p < 0.0001). Thus, only the daughters' urinary 8-oxodG and 8-oxoGua correlation was significantly different from 0.

Table 3 provides the analytic regression results of urinary 8-oxodG and 8-oxoGua on aMT6s/Cr alone and on aMT6s/CR, age, and BMI combined for mothers, fathers, and oldest daughters. (The results for all daughters, using a regression model which includes correlations between sisters, were similar to the results for the oldest daughters.) The aMT6s/Cr parameter estimates (regression line slope) and significance levels were nearly identical with and without age and BMI (or weight) in the model in each analysis. In the analyses for the mothers and fathers who were older than the oldest daughter (Table 4), the results were similar, except that specific p-values were lower.

**Table 3:** Regressions of Urinary Creatinine-Adjusted aMT6s, Age, and BMI on 8-oxodG and 8-oxoGua by Mother, Father, and Daughter

**DNA Repair Product**	**Subjects**	**Variables in Model**	**Slope (p-Value)**	**Slope (p-Value)**	**Slope (p-Value)**
			aMT6s/CR	Age	BMI

**8-oxodG**	**Mothers**	aMT6s/CR	-0.025 (0.012)	--	--
		
		(aMT6s/CR, Age, BMI)	-0.025 (0.02)	-0.008 (0.8)	0.051 (0.4)
	
	**Fathers**	aMT6s/CR	-0.01 (0.5)	--	--
		
		(aMT6s/CR, Age, BMI)	-0.01 (0.5)	-0.027 (0.6)	0.02 (0.8)
	
	**Daughters**	aMT6s/CR	-0.02 (0.27)	--	--
		
		(aMT6s/CR, Age, BMI)	-0.02 (0.14)	-0.008 (0.9)	-0.17 (0.12)

**8-oxoGua**	**Mothers**	aMT6s/CR	0.24 (0.17)	--	--
		
		(aMT6/CR s, Age, BMI)	0.23 (0.2)	-0.88 (0.09)	1.73 (0.12)
	
	**Fathers**	aMT6s/CR	-0.77 (0.07)	--	--
		
		(aMT6s/CR, Age, BMI)	-0.80 (0.063)	-0.86 (0.35)	2.9 (0.18)
	
	**Daughters**	aMT6s/CR	0.01 (0.95)	--	--
		
		(aMT6s/CR, Age, BMI)	0.02 (0.9)	1.27 (0.07)	-0.04 (0.7)

**Table 4:** Regressions of Urinary Creatinine-Adjusted aMT6s, Age, and BMI on 8-oxodG and 8-oxoGua by Mothers and Fathers Older than the Oldest Daughter

**DNA Repair Product**	**Subjects**	**Variables in Model**	**Slope (p-Value)**	**Slope (p-Value)**	**Slope (p-Value)**
			aMT6s/CR	Age	BMI

**8-oxodG**	**Mothers**	aMT6s/CR	-0.03 (0.006)	--	--
		
		(aMT6s/CR, Age, BMI)	-0.029 (0.009)	0.30 (0.5)	0.11 (0.097)
	
	**Fathers**	aMT6s/CR	-0.01 (0.8)	--	--
		
		(aMT6s/CR, Age, BMI)	-0.07 (0.7)	-0.007 (0.9)	0.06 (0.6)
	
**8-oxoGua**	**Mothers**	aMT6s/CR	0.16 (0.43)	--	--
		
		(aMT6/CR s, Age, BMI)	0.22 (0.26)	-1.03 (0.15)	1.97 (0.09)
	
	**Fathers**	aMT6s/CR	-0.94 (0.04)	--	--
		
		(aMT6s/CR, Age, BMI)	-0.99 (0.03)	-1.21 (0.27)	3.5 (0.13)

The primary multivariate analysis results are as follows:

1. among all mothers, there was

a. a statistically significant (p = 0.02) **decrease **in 8-oxodG as endogenous overnight urinary aMT6s/Cr **increased **for models with or without BMI or weight,

b. a marginally significant (p < 0.08) **increase **in 8-oxodG as weight **increased **(data not shown), but not BMI;

2. among the mothers older than the oldest daughter (age 51.6),

a. the significance levels of aMT6s/CR fell to 0.009 and 0.008 for 8-oxodG with BMI or weight in the model, respectively;

b. BMI was marginally **positively **associated (p = 0.097), while weight was significantly **positively **associated (p = 0.04; data not shown) with 8-oxodG;

3. among all fathers, there was

a. a marginally significant (p = 0.063 and 0.065 with BMI or weight in the model, respectively) **decrease **in 8-oxoGua as endogenous overnight urinary aMT6s/Cr **increased**;

4. among the fathers older than the oldest daughter (age 51.6),

a. the **inverse **association between 8-oxoGua and aMT6s/CR was statistically significant (p = 0.03 for either BMI or weight in the model);

5. among the oldest daughters, there was a. a marginally significant (p < 0.07 and < 0.08 for BMI and weight, respectively) **increase **in 8-oxoGua as age **increased**.

Table 5 provides the correlations and percent of variation explained (R^2^) between aMT6s/Cr and 8-oxodG (mothers) and 8-oxoGua (fathers). The percent of variance explained by aMT6s/Cr pertains to the univariate regression and is 12.4% and 8.4% for the mothers' 8-oxodG and fathers' 8-oxoGua, respectively.

**Table 5:** Correlations and R^2 ^for Urinary Creatinine-Adjusted aMT6s and 8-oxodG (Mothers) and 8-oxoGua (Fathers)

**Subject**	**Variables**	**Correlation** **(p-Value)**	**R**^2^ **(as percent)**
Mothers	aMT6s/CR 8-oxodG	-0.35 (0.01)	12.4

Fathers	aMT6s/CR 8-oxoGua	-0.29 (0.07)	8.4

**Figure 1:**
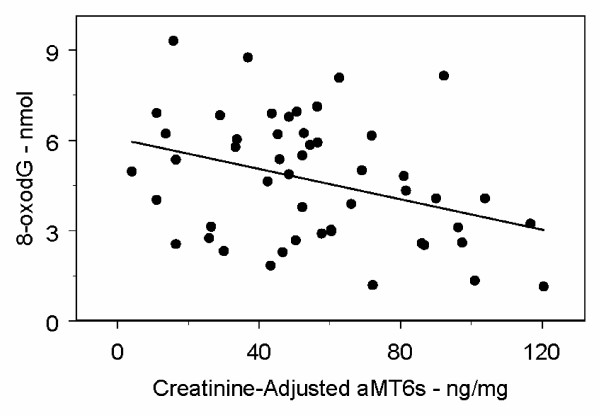
**Scatter Plot of Mothers' Total Nocturnal Urinary 8-oxodG (nmol) versus Total Nocturnal Urinary Creatinine-Adjusted aMT6s (ng/mg), with Regression Line**. The slope of the regression line is -0.025 (p = 0.01).

**Figure 2:**
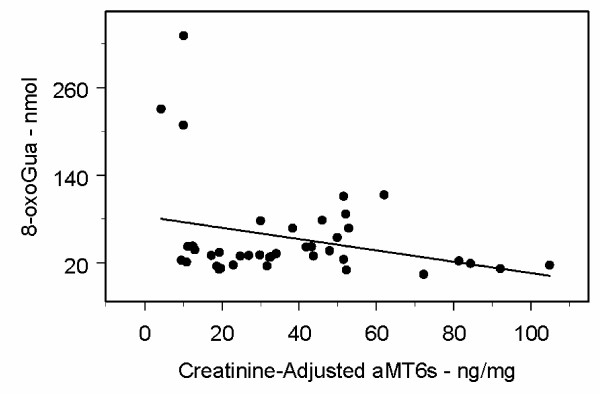
**Scatter Plot of Fathers' Total Nocturnal Urinary 8-oxoGua (nmol) versus Total Nocturnal Urinary Creatinine-Adjusted aMT6s (ng/mg), with Regression Line**. The slope of the regression line is -0.77 (p = 0.07).

Figure 1 and Figure 2 provide scatter plots of aMT6s/Cr and 8-oxodG (mothers) and 8-oxoGua (fathers) with the univariate regression lines added. Note that the differences between the slope estimates and p-values for aMT6s/Cr are minimal for the models with and without age and BMI (Tables 3 and 4) or age and weight. A straight line regression is appropriate for the mothers' 8-oxodG data, based on the scatter plot (Figure 1). However for the fathers' 8-oxoGua data, the scatter plot (Figure 2) shows there to be 3 very influential data points: the three points in the upper left-hand corner (low aMT6s/Cr and high 8-oxoGua). These 3 points represent 7.5% of the data and 2 outliers have already been eliminated, so it can certainly be argued that it is not appropriate to discard these three observations.

However, we conducted further linear regression analyses on the fathers' 8-oxoGua data transformed in four ways: natural logarithm, square root, reciprocal, and logistic. The logistic transformation is log [y/(1+y)], where 'log' is the natural logarithm function. These transformations are often used to reduce or eliminate the effect of outliers on statistical results. All four analyses, using the models with age and BMI or with age and weight, showed a marginally significant inverse association between the transformed 8-oxoGua and aMT6s/Cr values. The p-values were very close to the p-values for the untransformed regression model. For example for the models with age and BMI, the p-values were 0.063 (untransformed), 0.066 (log transform), 0.068 (square root transform), 0.069 (logistic transform), and 0.069 (reciprocal).

## Discussion and Conclusion

DNA damage, particularly damage to DNA repair genes, is considered one of the primary pathways to malignancy. Many cancers are thought to be associated with oxidatively damaged DNA, e.g., hormone-related cancers such as breast, ovarian and prostate cancer, lung cancer, and digestive system cancers (e.g., [16,17]). Estradiol is a major contributor to the production of ROS, including hydroxyl radicals, in ovarian and breast epithelial cells, which are the primary sites of these cancers [18,19]. ROS and reactive nitrogen species (RNS) are important causes of DNA damage and system malfunction [20]. Hydroxyl radicals are the most reactive and cytotoxic of the reactive oxygen species [21]. ^•^OH causes DNA base damage, particularly to guanine which is the DNA base most prone to oxidative damage [4,5]. The oxidation of guanine in DNA is premutagenic, leading to a G:C → T:A transversion mutation if not repaired, because an oxidized guanine preferentially pairs with adenine instead of cytosine [4]. Under the presumption that most DNA damage is repaired, the urinary levels of 8-oxodG and 8-oxoGua are indices of the rates of DNA damage. The higher either rate is, the higher is the risk of a G:C → T:A transversion mutation.

Melatonin is a hormone primarily produced by the pineal gland, mostly nocturnally. Melatonin appears to have many functions and has been evolutionarily conserved. "Normal" human production levels vary widely, by up to 2 orders of magnitude, and generally decline with age. Melatonin is found in both the cytoplasm and the nucleus of all cells in the human body and readily crosses the blood-brain barrier [2,3,22,23]. It functions both as a free molecule and when bound to any of its three receptors.

In addition to ^•^OH, melatonin also scavenges hydrogen peroxide (H_2_O_2_), nitric oxide (NO), peroxynitrite anion (ONOO^-^), hypochlorous acid (HOCl), and singlet oxygen (^1^O_2_) [24-28]. ^•^OH is produced at high levels by natural aerobic activity. Other radicals are also produced by various biological activities or result from certain environmental and lifestyle (e.g., smoking) exposures. Hydrogen peroxide does not appear to react directly with DNA [16], but does undergo chemical reactions within the cell nucleus which produces ^•^OH, e.g., reaction with Fe^+2^. On the other hand, ^1^O_2 _readily oxidizes guanine bases in DNA, but is neither as reactive and nor as cytotoxic as ^•^OH. ^•^OH appears not to be removed by antioxidative enzymes, but rather is detoxified by certain direct radical scavengers, such as melatonin [29].

Oxidatively damaged DNA is repaired by both base excision repair and nucleotide excision repair [7]. Many genes are involved in each repair pathway. Excision repair cross-complementing factor 6 (ERCC6) is important in both the BER and NER pathways. Human 8-oxoguanine DNA glycosylase 1 (hOGG1) is a BER enzyme responsible for identifying and assisting in the removal of oxidized guanine. Preliminary animal evidence indicates that melatonin may upregulate ERCC6 [30]. There is also preliminary cellular evidence that ERCC6 may, in turn, upregulate hOGG1 expression [31]. hOGG1 is inhibited by nitric oxide (NO) [32]. Melatonin generally inhibits nitric oxide availability and thus may also more directly upregulate hOGG1.

This report describes the results of what appears to be the first published study in humans of the relationships between endogenous melatonin production levels and levels of oxidatively damaged DNA. Because total overnight urinary aMT6s/Cr is highly correlated with total overnight melatonin production, the relationships between 8-oxodG, 8-oxoGua and aMT6s/Cr translate into similar relationships between 8-oxodG, 8-oxoGua and melatonin production.

Among the mothers, aged 43.5 - 80.7, higher levels of nocturnal melatonin production, as measured by total nocturnal urinary aMT6s/Cr, were associated with lower levels of total nocturnal urinary 8-oxodG. This translates into lower levels of oxidatively damaged guanine in DNA because nearly all such damage is repaired. Interestingly, fathers, aged 46.1 - 80.9, did not have an inverse relationship between total nocturnal urinary aMT6s/Cr and total nocturnal urinary 8-oxodG. Rather, they had a marginally significant inverse relationship between total nocturnal urinary aMT6s/Cr and total nocturnal urinary 8-oxoGua. (When the analysis was limited to fathers older than any daughter, the inverse relationship because statistically significant.) For the oldest daughters, aged 18.6 - 51.6, there was no relationship between total nocturnal melatonin production levels and total nocturnal urinary 8-oxodG or 8-oxoGua.

These data indicate that melatonin may influence the pathways used to repair oxidatively damaged guanine in DNA differently in older men than in older women. This possible difference between older men and older women is certainly intriguing. In addition, the mothers' nocturnal urinary levels of 8-oxodG, as a function of aMT6s/Cr, clearly appear to have a linear component with a negative slope (Figure 1). The fathers' nocturnal urinary levels of 8-oxoGua may have a non-linear relationship with aMT6s/Cr.

The percentages of variance in 8-oxodG (mothers) and 8-oxoGua (fathers) accounted for by aMT6s/Cr are not particularly large (12.4% and 8.4%, respectively). There are many other factors which influence the level of an individual's oxidatively damaged guanine in DNA. However, the level of production of melatonin appears to be a significant factor.

## Competing interests

The authors declare that they have no competing interests.

## Authors' contributions

ZD and ES contributed to all aspects of the design, implementation, and statistical analyses of the study. HEP and AW performed the 8-oxodG and 8-oxoGua assays. All authors contributed to the manuscript drafts, revisions and critical review of the final version.

## Pre-publication history

The pre-publication history for this paper can be accessed here:


